# Alterations in factors associated with diabetic retinopathy combined with thrombosis: A review

**DOI:** 10.1097/MD.0000000000034373

**Published:** 2023-08-04

**Authors:** Haiyan Wei, Xiaoping Xiao, Shuqin Zeng, Ye Liu, Xiaofang Liu, Tianyu Zeng, Pengxiang Xu, Wenyan Xia, Li Guo, Shihua Hong, Weiming Lv, Yijian Chen, Rong Xu

**Affiliations:** a The First Affiliated Hospital of Gannan Medical University, Ganzhou, P.R. China; b Ganzhou Key Laboratory for Drug Screening and Discovery, School of Geography and Environmental Engineering, Gannan Normal University, Ganzhou, P.R. China; c Provincial Key Laboratory of Low-Carbon Solid Waste Recycling, Gannan Normal University, Ganzhou, P. R. China.

**Keywords:** coagulation and fibrinolytic disorders, diabetic retinopathy, endothelial cells, inflammatory factors, platelets, thrombosis

## Abstract

Diabetic retinopathy (DR) is one of the most common and serious microvascular complications of diabetes mellitus, the incidence of which has been increasing annually, and it is the main cause of vision loss in diabetic patients and a common cause of blindness. It is now found that thrombosis plays a crucial role in the disease progression in DR patients, and the final vision loss in DR may be related to the occurrence of thrombosis in the retinal vessels, which is dominated by abnormal endothelial cell function, together with platelet dysfunction, imbalance of coagulation and fibrinolytic function, and related alterations of inflammatory factors leading to the main cause of thrombotic disease in DR patients. In this review, we examine the role between DR and thrombosis and the association of each factor, including endothelial dysfunction; platelet dysfunction; coagulation-fibrinolytic imbalance; and alterations in inflammatory factors.

## 1. Introduction

Diabetic retinopathy (DR) is one of common diabetic microangiopathies. DR is strongly associated with disease duration, glycemic changes, glycosylation end products and receptors, reduced insulin and C-peptide molecular genetic factors, obesity, cytokine levels, chronic inflammation, oxidative stress, hemodynamic abnormalities, abnormal coagulation mechanisms, and activation of protein kinase C signaling pathways.^[1–4]^ In the presence of these factors, the retinal microvascular basement membrane thickens and finally leads to thrombosis.

The stimulation of DR patients with chronic hyperglycemia and other factors in the body leads to the secretion of a large number of vasoactive substances by vascular endothelial cells, which in the presence of these active substances leads to an altered microcirculatory environment in the body and increases the risk of thrombosis.^[5]^ At the same time, the continuous high glucose state activates platelets, leading to platelet activation, enhanced platelet adhesion, increased blood viscosity, and changes in platelet-related factors, further providing a basis for thrombosis.^[6]^ The coagulation–fibrinolytic imbalance that accompanies DR patients in vivo leads to a hypercoagulable state of blood and an imbalance in the body’s coagulation and anticoagulation systems, which greatly exacerbates the risk of thrombosis.^[7]^ In addition, in patients with DR there are alterations of various inflammatory factors, which affect the endothelial function and aggravate platelet activation and coagulation–fibrinolytic imbalance, further leading to thrombosis.^[1]^

The relationship between diabetic retinopathy and thrombosis involves endothelial cell injury, platelet abnormality, coagulation-fibrinolysis imbalance, and changes of related inflammatory factors. The details are discussed as follows.

## 2. Endothelial cell injury in DR

In general, endothelial cells are capable of producing various vasoactive substances, such as thrombomodulin (TM), which exerts anticoagulant effects, to maintain the dynamic homeostasis of blood vessels and prevent thrombosis.^[5]^ When stimulated by infection, ischemia, hypoxia, acidosis and severe trauma, the normal function of endothelial cells is affected, pro-coagulant and anti-fibrinolytic substances, such as von Willebrand Factor (vWF) and plasminogen activator inhibitor-1 (PAI-1) are activated, while also inhibiting the production of anti-fibrinolytic and pro-fibrinolytic substances, such as tissue plasminogen activator (t-PA), leading to thrombosis.^[8,9]^

Endothelial cells also regulate vasodilation and contraction by secreting and synthesizing various vasoactive substances, including endothelial-derived relaxing factor, prostaglandin I2 (PGI2), endothelin-1, and a series of prostaglandin metabolites such as prostaglandin F2a.^[10,11]^ The synthesis of PGI2 is decreased and that of endothelin-1 (ET-1) is increased in diabetic patients, which is more significantly in DR.^[12,13]^

TM as a transmembrane glycoprotein synthesized by endothelial cells, exists on the surface of endothelial cells, and has a high affinity with thrombin, to play a clotting role.^[14]^ Previous studies have found that TM, a marker of vascular endothelial cell function impairment, can be detected in human blood and urine, and most of its detection is due to the escape of TM from the surface of vascular endothelial cells as a result of their dysfunction. In the normal population, plasma levels of TM are low, and with endothelial cell damage, the release of TM increases, which in turn leads to higher plasma TM levels, resulting in a hypercoagulable state of the blood.^[15]^ With the progression of the disease, extensive vascular endothelium damaged occurs in DR patients, and both systemic capillaries and retinal vessels may be blocked. A persistent state of hyperglycemic leads to significantly elevated plasma TM levels, leading to endothelial cell dysfunction. After leukocyte escape to the endothelial cell surface, their movement in the retina stops, resulting in capillary leakage, which leads to retinal circulation stagnation and increased permeability.^[16]^ Therefore, TM levels in plasma can be one of the markers for the presence or absence of damage to vascular endothelial cells in diabetic patients. It also helps to evaluate the endothelial cell damage and the extent of fundus retinopathy in diabetic patients.

vWF is a glycoprotein and one of the vascular endothelium-specific markers.^[17]^ It has 2 main effects: (1) vWF accelerates thrombosis by regulating platelet adhesion; (2) Carrying plasma factor VIII, maintains its activity and directly promotes thrombosis.^[18]^ Plasma vWF is also elevated in diabetic patients, so it is believed that vWF can effectively reflect endothelial cell damage in diabetic patients. Especially when hyperglycemia causes endothelial cell damage, a large amount of vWF is released into the blood, accelerating platelet adhesion and aggregation in the damaged blood vessels, leading to thrombosis. Therefore, vWF is considered to be a specific indicator of vascular endothelial function.^[19]^ vWF levels are significantly elevated in patients with DR.^[20]^ vWF is involved in the pathogenesis of DR by activating platelets and causing platelet aggregation, leading to a hypercoagulable state of circulating blood. As a biologically active form of factor VIII related antigen, it aggregates in large numbers when endothelium tissues are damaged, resulting in increased tissue factor concentration, activating the coagulation pathway, causing conformational changes in membrane glycoproteins IIb–IIIa. Activated platelets adhere to the damaged vessel wall through the bridging effect of vWF, directly affecting the formation of thrombus.^[21]^ It has also been reported that there is a significant correlation between decreased retinal blood circulation and the degree of increased vWF.^[22]^ Significantly elevated vWF levels have been detected in retinal tissues of patients with early diabetes mellitus, accompanied by reduced retinal blood flow and even slowed circulation, further causing ischemia and hypoxia in fundus-related tissues and possibly further aggravating the progression of DR. Therefore, detection of plasma vWF levels can be used as a marker to determine the impairment of vascular endothelial function and can effectively predict the progression of DR development.

t-PA and PAI-1 are two substances produced and secreted by vascular endothelial cells, which can effectively regulate the function of the fibrinolytic system, their dynamic balance plays a key role in preventing thrombosis and maintaining normal microcirculation.^[9]^ t-PA converts it to fibrinolytic enzymes by binding to fibrinogen (FIB), thus lysing the thrombus.^[23]^ PAI-1, the main inhibitor of t-PA, is negatively correlated with t-PA and can inhibit t-PA to a certain extent therefore, when t-PA and PAI-1 is imbalanced, fibrin inside and outside the blood vessels cannot be timely decomposed by the fibrinolytic system.^[24]^ High glucose can directly cause elevated levels of PAI-1, the overproduction of PAI-1 is the most typical phenotype of thrombosis in diabetes.^[25]^ If other metabolic disorders are present along with this condition, the probability of vascular lesions and thrombosis will be greatly increased. In DR patients, because of persistently elevated blood glucose and other factors, glycatied hemoglobin is accelerated, causing ischemia and hypoxia in vascular endothelial cells, thereby causing endothelial cell damage and dysfunction, resulting in a decrease in endothelial cell t-PA content. As the same time, the increased level of PAI-1 leads to an imbalance in the ratio of t-PA to PAI-1, which increase the incidence of thrombosis.^[26]^ In patients with DR, PAI-1 levels were found to gradually increase with the progression of the disease. Chronic hyperglycemia can stimulate endothelial cells to secrete large amounts of PAI-1, which will lead to hypercoagulation as a risk factor for thrombosis-associated disease. The severity of DR is related to the persistent hypercoagulable state and decreased fibrinolytic activity, the more severe the DR is associated with decreased fibrinolytic activity, which aggravates the development of retinopathy. As a result, on the one hand, the retinal barrier is destroyed resulting in a large amount of extravasation; on the other hand, large capillary occlusion lead to retinal ischemia and hypoxia, stimulating the release of vascular growth factor, producing a large number of neovascularization and promoting the development of DR. With the appearance of microangiopathy, fibrinolytic activity is further decreased, accompanied by deteriorating of coagulation function, long-term hypercoagulation and low fibrinolysis state leading to vascular stenosis and occlusion, insufficient blood perfusion, and small ischemic areas in the vessel wall, which cause secondary thrombosis and aggravated endothelial damage, thus forming an important basis for the development of diabetic microangiopathy. Therefore, the detection of serum t-PA and PAI-1 can more accurately determine the fibrinolytic state and the severity of complications in patients with DR changes providing an objective basis for clinical anticoagulation therapy and prevention of complications, which has important clinical significance.^[27]^

## 3. Abnormal platelet function in DR

Platelet aggregation and enhanced adhesion exist in diabetic patients. Platelet aggregation is associated with imbalance of thromboxane A2 (TXA2)/PGI2, which is mainly caused by oxygen free radicals on cell membranes, to produce lipid peroxides that damage biofilms and inhibiting prostaglandin synthase.^[28,29]^ This imbalance is increasingly evident in DR, suggesting that alterations in the concentration of the above substances are associated with microvascular diastolic abnormalities, microcirculatory disorders, and microvascular damage.^[6]^ Imbalance between PGI2 and TXA2 can promote microthrombosis and lead to retinopathy. Diabetes itself serve as an independent risk factor for vascular complications, and furthermore abnormalities of PGI2 and TXA2 will greatly increase the risk of retinopathy, so the judgment index for early diagnosis and prevention of DR can be determined by detecting TXA2 and PGI2 levels in diabetic patients, which has important clinical significance.

Thromboxane B2 (TXB2) and 6-keto-prostaglandin F1α (6-Keto-PGF1α) can indirectly reflect the TXA2 and PGI2 levels of patients, thereby predicting the thrombosis in patients. Under the effect of TXB2, blood vessels produced strong constriction, local tissue ischemia and hypoxia are significantly aggravated, resulting in increased retinal vascular permeability, protein leakage and eventually exudative retinopathy. With the development of DR, TXB2 increase and 6-keto-prostaglandin F1α decreased.^[30]^ Therefore, the measurement of plasma TXB2 levels provides insight into platelet activation and the severity of DR.

Platelet activation also accelerates the rapid translocation and expression of P-selectin, which can be stored in platelet α-particles translocated on the cell surface. P-selectin acts as an important hemostatic factor, activates the adhesion of platelets, neutrophils and monocytes, thereby improving the innate immune response of the organism, inducing its platelet binding and aggregation, the expression level is significantly increased after stimulating it.^[31]^ The level of soluble P-selectin in DR is significantly increased, which promotes leukocyte migration, aggregation and activation,^[32]^ reduces its deformability and retinal blood flow. Leukocytes have high cytoplasmic viscosity and larger diameter than capillaries, which will temporarily block microvessels, form retinal ischemic zone, cause the increase of peroxides and proteases, thus leading to endothelial cell dysfunction and injury. However, the damaged endothelial cells overexpress nitric oxide (NO) and lipid peroxides, which will affect the permeability of retinal blood vessels, leading to retinopathy. Therefore, detection of P-selectin levels on platelet surface can be used as a marker of platelet activation and an indicator to judge the severity of DR.

With further research, platelet involvement in the progression of DR activates the coagulation system of the body, resulting in a series of changes in indicators. First, resting platelets in circulating blood are stimulated to release cytoplasmic granule membrane glycoproteins, which fuse with the plasma membrane, and their morphology changes. The activated platelet surface glycoproteins undergo conformational rearrangement and release more coagulation factors, leading to microthrombosis and promoting the development of DR. Secondly, platelet aggregation function is significantly enhanced in DR patients, which may be related to the following factors: the persistent hyperglycemic state in diabetic patients leads to abnormal vascular endothelial function and increased secretion of coagulation factor VIII corresponding antigen by vascular endothelial cells, which promotes platelet aggregation and adhesion. Platelet aggregation function can respond to the degree of vascular damage and is positively correlated with blood glucose level. Platelet aggregation increases the release of PF-3, which feeds back to promote platelet agglutination, facilitate thrombosis and aggravate DR.

## 4. Imbalance between coagulation and fibrinolysis in DR

Some diabetic patients have retinopathy at the beginning of the disease, while some diabetic patients have been in a state of hyperglycemia for a long time without retinopathy. So it is considered that other factors besides hyperglycemia play an important role in DR. Blood viscosity is significantly increased in patients with DR.^[33]^ Due to the long-term damage of the vascular endothelium by the inflammatory response,^[34]^ which causes endothelial cell dysfunction and secretion of a large number of cytokines, resulting in increased levels of endogenous and exogenous coagulation factors activated partial thromboplastin time (APTT) and prothrombin time (PT). The activation of platelets and the coagulation system leads to a hypercoagulable state of blood and an imbalance in the coagulation and anticoagulation systems of the body, thus increasing the risk of thrombosis.^[7,35]^

Fibrinogen (FIB) is the most abundant coagulation factor in the blood. It is a macromolecular glycoprotein that is indispensable in the human coagulation system and has a role in maintaining the coagulation and anticoagulation homeostasis.^[35]^ The possible reasons why FIB promotes thrombosis include: increasing plasma and whole blood viscosity; and altering the shear force of vascular endothelial cells. In addition, extensive microvascular endothelial cell injury stimulates platelet aggregation and releases cytokines, which can promote endothelial cells synthesis and release of plasminogen activation inhibitors, ultimately leading to reduced plasma FIB degradation. Thereby, increasing the probability of local thrombosis. Studies of fibrin clot structure in DR patients have shown that FIB is glycosylated in vivo, resulting in changes in fibrin clot structure, decreased permeability, reduced fibrinolysis, resulting in increased fibrin in vivo, increased blood viscosity, and poor blood fluidity, the formation of a hypercoagulable state.^[36]^ Upregulation of FIB levels causes many pathological changes that mediate the progression of DR. In DR the increased FIB levels accelerate the pathological state caused by hyperglycemic pro-inflammatory and pro-angiogenic factors, which is manifested as a hypercoagulable state and increased blood viscosity, increased with prolonged disease and ocular exacerbation. In addition, under other stimuli, such as the stimulation of angiogensis reaction, endothelial permeability will increase, reactive oxygen species will be generated, and the stimulation of various inflammatory factors and aggregation of platelets will lead to the imbalance of coagulation–fibrinolytic system, and increasing the risk of thrombosis.^[21]^

APTT is an important indicator to evaluate the endogenous coagulation system. In certain diseases, APTT values are shortened when the endogenous coagulation system is abnormal in function, such as thrombophilia and early disseminated intravascular coagulation.^[37]^ When DR occurs, due to factors such as hyperglycemia and hyperlipidemia in the body lead to a chronic inflammatory response in the vascular endothelium of the retinal vessels, resulting in anticoagulation dysfunction and platelet activation, thus destorying the dynamic balance between coagulation-fibrinolytic systems, and causing thrombosis. A reduced APTT means that the thrombogenic mechanism has been activated in such patients and puts their blood in a hypercoagulable state. Therefore, the analysis of coagulation and fibrinolytic function indicators can predict thrombosis and its severity, clarified the severity of DR, and provided a valuable basis for clinical anticoagulation treatment.

## 5. Changes in inflammatory cytokines in DR

Normal endothelial cells can release PGI2 and NO through TM and extracellular, thus play a role in inhibiting thrombus. However, in DR, due to increase of inflammatory response, leukocytes activate a large number of cytokines, such as interleukin-1 (IL-1), interleukin-6 (IL-6), and tumor necrosis factor-α (TNF-α).^[38]^ These cytokines can cause dysfunction of vascular endothelial cells. In addition to the disappearance of their own antithrombotic effect, dysfunctional endothelial cells can overexpress tissue factor (TF) and vWF, which contributes to the activation of coagulation factors and eventually causes thrombosis. When the inflammatory response is enhanced, the activity of coagulation factors in vivo increase,^[39]^ causing the damaged vascular wall cells to produce protein disulfide isomerase (PD), further activates TF activity and initiates exogenous coagulation pathways.^[40,41]^ In addition, inflammation promotes the production of fibrin and factor VIII, which causes the release of neutrophil extracellular traps (NETs) from neutrophils, promotes the production of thrombin, thus promoting an increase in coagulation factors in the circulation. The combined action of inflammation and coagulation factors further exacerbates the inflammatory response in the vessel wall through a common activation pathway, leading to thrombosis. Studies have reported that the occurrence of DR is closely related to pro-inflammatory factors. The production of reactive oxygen species, pro-inflammatory factors, and certain growth factors can cause damage to the retina.^[1]^ The levels of cytokines such as TNF-α, IL-6 and IL-8 were detected to be elevated in the vitreous fluid of patients with DR,^[1]^ Elevated levels of these inflammatory markers can further lead to thrombosis by activating the coagulation system. Therefore, detection markers of inflammation in patients with DR, combined with coagulation indicators can predict the risk of thrombosis.

## 6. Summary

**Figure 1.**
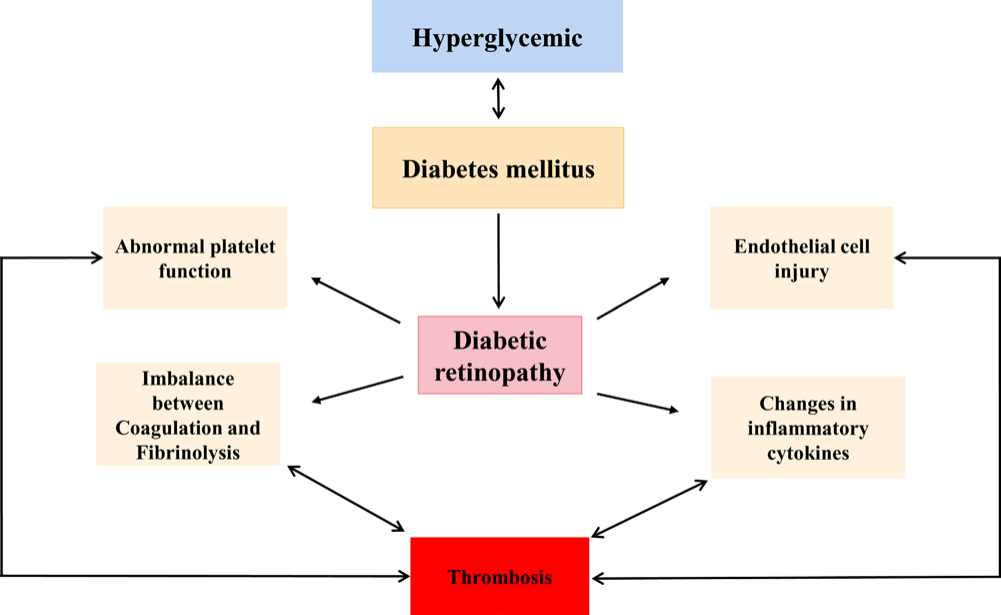
Diabetic retinopathy is one of the common chronic complication of DM, and is currently the main cause of vision loss in the population. The development of DR is often accompanied by damage to vascular endothelial cells, platelet abnormalities, imbalance in coagulation and fibrinolysis, and increased inflammatory factors, resulting in microcirculatory blockage, local tissue ischemia and hypoxia, and consequently thrombosis. DM = diabetes mellitus, DR = diabetic retinopathy.

In summary, the progression of DR is often accompanied by vascular endothelial cells damaged, platelet abnormalities, coagulation-fibrinolytic imbalance, and increased inflammatory factors. Eventually, these factors lead to microcirculatory blockage, local tissue ischemia, and thrombosis (Fig. 1). Therefore, real-time monitoring of the changes of the factors and effective interventions can reverse and possibly avoid further aggravation of the disease, also provide new potential therapeutic targets for early prevention, early diagnosis and early anticoagulation of DR combined with thrombosis.

## Author contributions

**Conceptualization:** Xiaoping Xiao, Shuqin Zeng, Ye Liu, Tianyu Zeng, Pengxiang Xu, Wenyan Xia, Li Guo, Shihua Hong, Yijian Chen.

**Formal analysis:** Xiaofang Liu.

**Supervision:** Weiming Lv, Rong Xu.

**Visualization:** Ye Liu.

**Writing – original draft:** Haiyan Wei.
